# Healthcare Costs and Healthcare Utilization Outcomes of Vitamin D3 Supplementation at 5000 IU Daily during a 10.9 Month Observation Period within a Pragmatic Randomized Clinical Trial

**DOI:** 10.3390/nu15204435

**Published:** 2023-10-19

**Authors:** Patrick J. LaRiccia, Teresa Cafaro, Dibato John, Noud van Helmond, Ludmil V. Mitrev, Brigid Bandomer, Tracy L. Brobyn, Krystal Hunter, Satyajeet Roy, Kevin Q. Ng, Helen Goldstein, Alan Tsai, Denise Thwing, Mary Ann Maag, Myung K. Chung

**Affiliations:** 1 Won Sook Chung Foundation, Moorestown, NJ 08057, USA; lariccip@pennmedicine.upenn.edu (P.J.L.); cafaro-teresa@cooperhealth.edu (T.C.); bbandomer@wonsookchungfoundation.org (B.B.); brobyntl@rowan.edu (T.L.B.); ng-kevin@cooperhealth.edu (K.Q.N.); pharmhand95@gmail.com (H.G.); thewinger@aol.com (D.T.); mam43@aol.com (M.A.M.); dockyu@gmail.com (M.K.C.); 2 Center for Clinical Epidemiology and Biostatistics Perelman School of Medicine, University of Pennsylvania, Philadelphia, PA 19104, USA; 3 Department of Anesthesiology, Cooper University Health Care, Camden, NJ 08103, USA; vanhelmond-noud@cooperhealth.edu; 4 Cooper Research Institute, Cooper University Health Care, Camden, NJ 08103, USA; john-epoh-dibato@cooperhealth.edu (D.J.); hunter-krystal@cooperhealth.edu (K.H.); 5 Cooper Medical School of Rowan University, Camden, NJ 08103, USA; roy-satyajeet@cooperhealth.edu (S.R.); alantsai57@gmail.com (A.T.); 6 The Chung Institute of Integrative Medicine, Moorestown, NJ 08057, USA; 7 Rowan University School of Osteopathic Medicine, Stratford, NJ 08084, USA; 8 Division of General Internal Medicine, Cooper University Health Care, Camden, NJ 08103, USA; 9 Division of Infectious Disease, Cooper University Health Care, Camden, NJ 08103, USA; 10 Department of Family Medicine, Cooper University Health Care, Camden, NJ 08103, USA

**Keywords:** vitamin D, vitamin D3, costs, utilization, hospitalizations, emergency room visits, intensive care unit admissions, healthcare, healthcare workers

## Abstract

Vitamin D insufficiency has been linked to multiple conditions including bone disease, respiratory disease, cardiovascular disease, diabetes, and cancer. Observational studies indicate lower healthcare costs and healthcare utilization with sufficient vitamin D levels. The secondary aims of our previously published pragmatic clinical trial of vitamin D3 supplementation were comparisons of healthcare costs and healthcare utilization. Comparisons were made between the vitamin D3 at 5000 IU supplementation group and a non-supplemented control group. Costs of care between the groups differed but were not statistically significant. Vitamin D3 supplementation reduced healthcare utilization in four major categories: hospitalizations for any reason (rate difference: −0.19 per 1000 person-days, 95%-CI: −0.21 to −0.17 per 1000 person-days, *p* < 0.0001); ICU admissions for any reason (rate difference: −0.06 per 1000 person-days, 95%-CI: −0.08 to −0.04 per 1000 person-days, *p* < 0.0001); emergency room visits for any reason (rate difference: −0.26 per 1000 person-days, 95%-CI: −0.46 to −0.05 per 1000 person-days, *p* = 0.0131; and hospitalizations due to COVID-19 (rate difference: −8.47 × 10^−3^ per 1000 person-days, 95%-CI: −0.02 to −1.05 × 10^−3^ per 1000 person-days, *p* = 0.0253). Appropriately powered studies of longer duration are recommended for replication of these utilization findings and analysis of cost differences.

## 1. Introduction

Healthcare costs and healthcare utilization continue to rise [1,2,3,4]. Likewise, the expense of medical insurance has become prohibitive for many families and small businesses [5]. According to a survey in 2009, US workers reported paying double their insurance premiums over the preceding decade. Employees’ premium costs rose from USD 1543 on average in 1999 to USD 3515 in 2009 [6]. As healthcare expenditures increase, employers try to lessen their burden by sharing this increase with employees. Employees are seeing increases in their premiums, copayments, and deductibles, which must be paid out of their pocket before coverage can begin [7]. Even hospitals and physicians are not protected from the economic pressures; institutions are paying the bills for 20 to 25% of total medical care given, due to individuals who are uninsured [5]. Changes are needed in our healthcare practice that will reduce healthcare costs and healthcare utilization. Less costly treatments and increased prevention are needed.

Vitamin D supplementation can be considered in light of previous studies linking vitamin D insufficiency to multiple diseases. **Skeletal diseases** such as osteoporosis and painful osteomalacia in adults, and the inability of children to attain optimal bone mass are often the result of inadequate vitamin D levels [8]. The tissues that respond to the active form of vitamin D, 1,25(OH)2D, and help to promote mineral homeostasis and bone health are the small intestine, kidneys, and bone tissues [8]. In addition to its effects on bone health, vitamin D has also been linked to **respiratory diseases**. Supplementation with vitamin D has been reported to prevent influenza-like illness and acute respiratory tract infections [9,10]. Inadequate blood concentrations of vitamin D are associated with inappropriate activation of the renin–angiotensin–aldosterone system (RAAS) [11]. Vitamin D also enhances the production of human cathelicidin, a peptide known for its antimicrobial effects, and it controls the cytokine responses and T helper cell balance which helps in favorably modulating the adaptive immune system [12]. Gilbert et al. report there are strong data supporting the association between low vitamin D levels and chronic lung diseases [13]. They further explain that lung tissue inflammation and disrupted pulmonary cell movement have been associated with the presence of matrix metalloproteinase enzymes (MMPs). They described an inverse relationship between vitamin D levels and circulating MMPs (MMP-9) in a small population of healthy adults. This inverse relationship prompted the researchers to supplement a subset of subjects with vitamin D, which significantly reduced MMP-9 levels [13].

Low vitamin D levels increase the risk for **severe coronavirus 2** (SARS-CoV-2) [14]. SARS-CoV-2 enters human cells through cell surface angiotensin-converting enzyme 2 (ACE-2) receptors found on the epithelial lining of the lungs, gut, and mucus membranes [15,16]. ACE-2 receptors are also found on the “smooth cells of the blood vessels, heart (epicardia, adipocytes, fibroblasts, myocytes, coronary arteries), lung (macrophages, bronchial and tracheal epithelial cells, type 2 pneumocytes), brain, testis, and on tubular epithelial cells of kidney” according to Shirbhate et al. [17]. Vitamin D activity on ACE-2 receptors may be useful in the treatment of SARS-CoV-2 [18].

Vitamin D insufficiency is also associated with the risks of **cardiovascular disease** and hypertension [19]. Judd et al. propose the protective mechanisms of vitamin D on the cardiovascular system include “effects on the renin-angiotensin system, on glycemic control, inflammatory cytokines, direct effects on the vasculature and regulation of parathyroid hormone (PTH) levels, and calcium deposition in vascular smooth muscle” [19].

Vitamin D deficiency could be associated with the development of **diabetes** type I and II according to Martin et al. [20]. This group reported findings from the National Health and Nutrition Examination Survey (NHANES) III study between 1988 and 1994 that indicated an inverse association between diabetes type II prevalence and low vitamin D levels. In addition, the group described a cohort study in children from Northern Finland who were supplemented with 2000 IU vitamin D. The supplemented group of children was found to be 80% less likely to acquire type I diabetes [20]. Treatment with vitamin D has been shown to improve insulin resistance and glucose tolerance [20]. The insulin-secreting beta cells of the pancreas contain vitamin D receptors [20]. It has been reported that mouse beta cells with malfunctioning vitamin D receptors adversely affect the insulin secretory response [21]. Vitamin D deficiency has been shown to reduce insulin secretion, whereas vitamin D repletion has exhibited improved insulin secretion in animals [20].

Associations between low vitamin D levels and **cancer** have been reported by Edlich et al. [22]. The authors described the inverse association between breast cancer incidence and vitamin D levels. They also reported vitamin D deficiencies found among prostate, ovarian, and multiple myeloma cancer patients. The authors further summarized the photobiological mechanisms that produce previtamin D3 from 7-dehydrocholesterol in human skin. They reported the optimal wavelength of solar radiation was between 295 and 300 nm for the conversion to previtamin D3. When human skin was exposed to similar wavelengths of natural sunlight and simulated sunlight, 65% and 20%, respectively, of the original 7-dehydrocholesterol in the skin was converted to previtamin D3. The authors asserted natural sunlight and its spectral quality greatly affect the photochemistry of 7-dehydrocholesterol. After previtamin D3 is produced, it is then converted to vitamin D3 via an isomerization reaction induced thermally in the skin [22]. Edlich et al. explain over 1000 genes in various cell types throughout the body are thought to be regulated by vitamin D. In the cell nucleus, a complex is formed when the vitamin D molecule binds to a vitamin D receptor. This complex further binds to the retinoid-x receptor (RXR) which then binds to a region on the deoxyribose nucleic acid strand called the vitamin D response element. This activates gene transcription to occur with the subsequent manufacturing of the encoded protein. The authors conclude, “this broad-acting gene switch plays a major role in the proteins regulated by vitamin D” [22]. In animal experimental studies, Dusso et al. explained the active 1,25(OH)2D suppresses the RAAS, modulates immune cell function, and suppresses abnormal cell proliferation [23].

Zhang et al. reported in a meta-analysis of seven cohort studies totaling 4204 participants that low levels of vitamin D were associated with increased hospital mortality in critically ill patients. This finding was consistent in all strata of subgroup analyses [24].

Liu et al. analyzed the 2001–2010 National Health and Nutrition Examination Survey (NHANES) of a representative U.S. adult sample of 26,010 participants. 25-hydroxy vitamin D (25(OH)D) measurements were collected. Vitamin D deficiency (VDD) was defined as 25(OH)D less than 50 nanomoles/liter (nmol/L) while vitamin D insufficiency (VDI) was defined as 25(OH)D levels ≥50 nmol/L and ≤75 nmol/L. The analysis found a prevalence of VDD of 28.9% and a prevalence of VDI of 41.4% thus indicating low vitamin D levels in 70.3% of the U.S. population. There was a greater prevalence of VDD in adults who were black, current smokers, infrequent drinkers, less educated, obese, physically inactive, and poor. Non-Hispanic blacks had the highest prevalence of low vitamin D among racial groups. After adjustment for other potential factors, old adults were 63% more likely to have VDD and 46% more likely to have VDI than young adults. Among the independent predictors, being a minority was the strongest indicator for VDD and VDI. Obese adults had a 3.09 times higher prevalence of VDD and a 1.88 times higher prevalence of VDI than non-obese adults [25]. African Americans are particularly at risk for vitamin D deficiency because darker skin limits the penetration of UVB in the skin. The 2011–2021 NHANES data revealed that 39% of African Americans and 22% of Mexican Americans and Hispanics have vitamin D deficiency [26].

In addition to correlations between medical disorders and insufficient vitamin D levels, there appears to be a connection between vitamin D insufficiency and increased healthcare costs and healthcare utilization. A Veterans Affairs (VA) facility in Northeastern Tennessee performed a retrospective electronic chart review of all patients seen between 2005 and 2007. Out of 42,588 patients seen at the VA center during this period, 886 had records of 25(OH)D. A total of 40.5% of the sample population (*n* = 886) was vitamin D deficient (<20 ng/mL). Researchers obtained and analyzed vitamin D levels, healthcare costs, and healthcare services utilized over the 2 years preceding initial vitamin D level collection. They found vitamin D deficiency was closely linked to increased healthcare costs and healthcare utilization in veterans: overall cost, emergency room visits, clinic visits, inpatient services, and hospital stays were greater in the vitamin D deficient patients when compared to those with adequate levels [27].

Similarly, vitamin D deficiency was associated with increased healthcare costs in two separate retrospective studies at six Veterans Affairs Medical Centers in the Southeastern United States [28,29]. The first VA study observed vitamin D testing patterns and their links to medical costs from 2004 to 2008. An analysis of 15,340 patient records with available vitamin D data revealed that vitamin D-deficient patients had significantly higher total outpatient costs as compared to non-vitamin D-deficient patients [28]. Likewise, in the highest inpatient cost categories, laboratory and pharmacy, data showed doubled costs among patients who were vitamin D deficient compared to those who were not [28]. One VA center with an increased minority presence was found to have high deficiency levels but lower vitamin D testing patterns than that of other VA sites [28]. The other study conducted among six VA centers focused on rurality status and its association with healthcare costs from 2003 to 2009. A review of 9396 veteran records was grouped by rurality status and found the rural and large metro (inner city) areas had higher vitamin D deficiency rates and higher medical costs/service utilization than their urban counterparts [29]. Those who lived in large metro areas were 49% more likely to be vitamin D deficient, while those in rural areas were 20% more likely to be deficient in vitamin D when compared to urban residents [29].

In a community hospital, a prospective observational study was conducted among 258 patients who were consecutively admitted to the surgical intensive care unit (ICU). Vitamin D levels were collected on all patients within 24 h of being admitted to the ICU. The group categorized vitamin D deficiencies as follows: severe was less than 13 ng/mL; moderate was 14 to 26 ng/mL; mild was 27 to 39 ng/mL; and normal was greater than 40 ng/mL. Only 1.2% of the patients had normal vitamin D levels at admission/baseline. Results showed a correlation of vitamin D deficiency categories from normal to severe with increasing costs, increasing lengths of stay, and increasing mortalities [30]. In another community hospital study, 565 patients were divided into two groups: those with vitamin D levels less than 18 ng/mL compared to those with vitamin D levels above 18 ng/mL. Patients with vitamin D levels less than 18 ng/mL had higher hospital ward costs and higher ICU costs. The deficient group also had more frequent myocardial infarctions, ventilator-associated pneumonia, and longer hospital ward and ICU stays [31]. Two German independent population-based cohort studies (*n* = 7217 total) assessed vitamin D levels and health care costs and found vitamin D deficiency was associated with increased total average annual costs. In the two studies, greater than 60% and approximately 40% of the sample populations were vitamin D deficient (25OHD < 20 ng/mL) or severely deficient (25OHD < 10 ng/mL) at baseline. The authors concluded, “non-linear associations between the 25OHD concentration and inpatient costs and hospitalization were detected: participants with 25OHD concentrations of 5, 10 and 15 ng/mL had 226.1%, 51.5% and 14.1%, respectively, higher inpatient costs than those with 25OHD concentrations of 20 ng/mL (overall *p*-value = 0.001) in multivariable models” [32].

Based on our literature review and clinical experience, we postulated that people with sufficient vitamin D levels would have lower healthcare costs and less healthcare utilization than those with lower vitamin D levels. Our previously published article [10] demonstrated that vitamin D3 supplementation at 5000 IU/day reduces influenza-like illness in hospital workers [10]. In this companion article, we describe the results of the secondary aims of this study which were to assess healthcare costs and healthcare utilization outcomes [10].

## 2. Materials and Methods

Details of our pragmatic randomized clinical trial examining the effects of daily intake of 5000 IU of vitamin D3 on the incidence of influenza-like illness in healthcare workers (Clinicaltrials.gov: NCT04596657) have been published earlier [10]. Included in that publication are the CONSORT flow diagram and CONSORT checklist. The local Institutional Review Board approved the study (IRB #20-455). Healthcare workers who actively participated in the study provided informed consent. Here, we describe our methods of data acquisition and analysis of our secondary aims of comparing healthcare costs and healthcare utilization in the control and intervention groups.

### 2.1. Subjects

Employees of an inner-city university hospital who were at least 18 years of age were eligible to participate; exclusion criteria consisted of conditions or medications and supplements that could increase health risk by receiving vitamin D supplementation (Table 1). All subjects analyzed in the costs and utilization part of the study were insured for their healthcare through the university hospital. Subjects who were not insured through the university hospital healthcare plan were not included in the analyses as their healthcare costs and utilization records were not available. In addition, subjects in the passive control group were those who voluntarily completed a survey that included their informed consent, demographics, and medical history which were used for comparison with the intervention group’s demographics and clinical characteristics.

### 2.2. Vitamin D

A daily dose of 5000 IU is required to attain normal serum 25(OH)D concentrations in individuals who have concentrations below 55 nmol/L at baseline without supplementation [33]. Furthermore, in the state of New Jersey where this study was conducted, 28% of adults over the age of 20 are obese [34], and obese individuals require 2–3 times the normal dose of vitamin D supplementation for vitamin D deficiency [35]. The protective effect of vitamin D supplementation on acute respiratory tract infections that was found in systematic reviews in individuals without particularly low serum concentrations of 25(OH)D supports providing supplementation of vitamin D3 to individuals who may not be deficient in serum vitamin D by current clinical standards [9,36,37].

Considering the excellent safety profile of vitamin D3 at a dose of 5000 IU/day [38,39,40,41,42], we did not include laboratory testing or other clinical interventions in our procedures unless clinically indicated. Subjects were monitored via monthly surveys that queried subjects on symptoms of hypercalcemia and nephrolithiasis, which included 15 symptoms [43]. See [10] for more details on our main outcomes study.

### 2.3. Intervention and Control Groups

As indicated above the intervention group received 5000 IU of vitamin D3 per day for 9 months. The passive control group received no specific instructions and was followed from the start of the study until the last participant of the intervention group completed 9 months of vitamin D3 supplementation.

### 2.4. Observation Periods

The observation periods were the sum of individually calculated, de-identified subject data for each group. The individual intervention subjects’ observation period began on their date of first dose (plus sixty days) or the date their insurance coverage began, whichever was later; their observation period ended on the date of their last dose or the date their insurance coverage was terminated, whichever was earlier.

The individual control subjects’ observation periods began on the date of the first *intervention* subject’s first dose (plus sixty days), or the date insurance coverage began for the control subject, whichever was later; it ended on the date of the last *intervention* subject’s last dose or the date insurance coverage was terminated for the control subject, whichever was earlier.

Sixty days were added to the date of first dose of vitamin D3 for the intervention group subjects as this is the time period known to achieve therapeutic vitamin D blood levels [33]. The first intervention subject’s first dose plus sixty days was 2 January 2021. The last intervention subject’s last dose was 23 November 2021. The overall time span observed for all subjects combined was 10.9 months (326 days). The person-time denominators for the control and intervention groups were 590,348 and 37,935 person-days, respectively.

### 2.5. Data Acquisition

De-identified data on healthcare costs and healthcare utilization was obtained from the administrators of the university hospital employee insurance plan.

### 2.6. Measurements and Statistical Analysis

#### 2.6.1. Demographics and Clinical Characteristics

Demographics and comorbidity data were collected from both groups via survey. All subjects in the passive control group were invited to voluntarily complete a survey that included their informed consent, demographics, and medical history which were used for comparison with the intervention group’s demographics and clinical characteristics.

Descriptive statistics were used to describe the demographics and comorbidities of the intervention and control groups. To provide an objective means to identify meaningful differences in demographic and clinical characteristics between the intervention and control groups, we used standardized mean differences with a cutoff of 20% or 0.20 [44,45,46].

#### 2.6.2. Healthcare Costs

Healthcare costs for the control and intervention groups were determined for six categories including total billed charges for any reason; cost of hospitalizations due to COVID-19; cost of ICU admissions due to COVID-19; cost of ventilator use due to COVID-19; medical pharmacy prescription costs for any reason; and freestanding prescription costs for any reason. All costs were determined by the billed charges for each category. The mean cost per person-day (standardized mean) was calculated for each category. Differences in standardized means between control and intervention groups were assessed using Wilcoxon rank sum tests to determine statistical significance. The alpha level was set at 0.05. All analyses were conducted using SAS 9.4 (SAS Institute, Inc., Cary, NC, USA).

#### 2.6.3. Healthcare Utilization

Healthcare utilization was determined for fifteen categories: (1) number of hospitalizations for any reason; (2) number of ICU admissions for any reason; (3) number of emergency room visits for any reason; (4) number of hospitalizations due to COVID-19; (5) number of ICU admissions due to COVID-19; (6) all other outpatient units for any reason; (7) number of urgent care visits for any reason; (8) number of primary care physician units for any reason; (9) number of nurse practitioner units for any reason; (10) all other professional units for any reason; (11) number of medical pharmacy units for any reason; (12) number of freestanding prescriptions for any reason; (13) number of ventilator use for any reason; (14) number of ventilator use due to COVID-19; and (15) number of deaths for any reason.

Incidence rates (number of events per person-days) for all utilization categories were calculated and compared between control and intervention groups using count models (Poisson (P), negative binomial (NB), and zero-inflated negative binomial (ZINB)) with person-days used at offset. The model with the smallest Akaike Information Criterion (AIC) and Bayesian Information Criterion (BIC) values was chosen for each event. All analyses were conducted using SAS 9.4 (SAS Institute, Inc., Cary, NC, USA) and conclusions were made at 5% significance level. The above statistical methods were used because of the frequent occurrence of a small number of outcome events in the various utilization categories.

## 3. Results

### 3.1. Subjects

The sample size for the intervention group and passive control group was 196 and 1958, respectively, as this was the number of study subjects who were insured by the university hospital.

### 3.2. Demographics and Clinical Characteristics

Demographics and clinical characteristics were similar in the control and intervention groups, Table 2. We compared intervention group subjects (196) to control group subjects who voluntarily provided their demographic and comorbidity data via a survey (444 out of 1958). We found no relevant differences between the groups for a range of demographic and clinical characteristics, except for age, Hispanic or Latino ethnicity, and Not Hispanic or Latino ethnicity, each of which was slightly above the predefined standardized difference threshold of 0.20 (the standardized difference was 0.23 in each case).

### 3.3. Healthcare Costs

The total billed costs in the control group were USD 41,109,649.83 while the total billed costs in the intervention group were USD 2,318,500.31. The person-time denominators for the control and intervention groups were 590,348 and 37,935 person-days, respectively.

Three of the six measured parameters indicated lower costs in the intervention group. Two parameters indicated less costs in the control group. One parameter indicated no difference at all. There were no statistical differences in any of the cost comparisons. There was a statistical trend in the free-standing pharmacy cost comparison indicating less cost for the control group. See Table 3.

### 3.4. Healthcare Utilization

Four of the fifteen measured parameters comparing the control group with the intervention group showed a statistically significant difference indicating lower healthcare utilization in the intervention group. The four parameters were: hospitalizations for any reason (rate difference: −0.19 per 1000 person-days, 95%-CI: −0.21 to −0.17 per 1000 person-days, *p* < 0.0001); ICU admissions for any reason (rate difference: −0.06 per 1000 person-days, 95%-CI: −0.08 to −0.04 per 1000 person-days, *p* < 0.0001); emergency room visits for any reason (rate difference: −0.26 per 1000 person-days, 95%-CI: −0.46 to −0.05 per 1000 person-days, *p* = 0.0131; and hospitalizations due to COVID-19 (rate difference: −8.47 × 10^−3^ per 1000 person-days, 95%-CI: −0.02 to −1.05 × 10^−3^ per 1000 person-days, *p* = 0.0253).

There was a trend toward statistical significance for the number of urgent care visits for any reason and the number of ICU admissions due to COVID-19, indicating fewer in the intervention group. Five parameters indicated greater utilization in the intervention group but were not statistically significant: all other outpatient units for any reason, number of primary care physician units for any reason, all other professional units for any reason, number of medical pharmacy units for any reason, and number of freestanding prescriptions for any reason. One of the four remaining comparisons, number of nurse practitioner units for any reason, showed decreased utilization in the intervention group without statistical significance. See Table 4. The last three comparisons, number of ventilator use for any reason, number of ventilator use due to COVID-19, and number of deaths for any reason, showed no difference at all (all entries were zero and thus are not listed in Table 4).

### 3.5. Cost of Vitamin D3

Had subjects paid for their vitamin D3 they would have spent approximately USD 0.32 per day or USD 9.48 per month.

## 4. Discussion

### 4.1. Principal Findings

The insurance claims data of subjects randomized to the vitamin D3 intervention arm and of those subjects randomized to the passive control group in a pragmatic randomized clinical trial was examined for healthcare costs and healthcare utilization. Six healthcare cost parameters and fifteen healthcare utilization parameters were evaluated. In the vitamin D3 intervention group, there were non-statistically significant decreases in: total billed charges for any reason; cost of hospitalization due to COVID-19; and cost of ICU admissions due to COVID-19. Utilization claims data indicated four areas in which the intervention group showed statistically significant decreases in healthcare utilization: number of hospitalizations for any reason; number of ICU admissions for any reason; number of emergency room visits for any reason; and number of hospitalizations due to COVID-19.

The healthcare utilization results of this study that took place in an east coast inner-city university hospital in the United States concur with the studies that were performed in Veterans Administration (VA) Medical Centers in Northeastern Tennessee, VA Medical Centers in the Southeastern US, two US community hospitals, and two independent population studies in Germany [27,28,29,30,31,32] as mentioned above. However, while directional trends in costs were similar in the present study, there were no statistically significant cost differences. A total sample size of 6098 would have been required to provide sufficient power to detect a mean difference of USD 8.04/person-day. Our total sample size (both groups combined) was 2154. Had our study been longer, it is possible that there would have been greater cost differentials. The above studies were cross-sectional and retrospective. Our study was a prospective pragmatic clinical trial, which when combined with the other studies, indicates that despite differing populations, geographic locations, and methodology there is a degree of convergence toward healthcare utilization reduction in the vitamin D3 sufficient groups.

### 4.2. Mechanisms of Vitamin D Action

It is well-established that vitamin D deficiency is a worldwide problem [12] that has been shown to have unfavorable effects on multiple body systems. Vitamin D is utilized in multiple physiological mechanisms with diverse effects on body functions and systems. Vitamin D impacts the renin–angiotensin–aldosterone system which is involved with blood pressure regulation and volume homeostasis, and low levels are linked to cardiovascular disease [11,19]. Vitamin D enhances the production of cathelicidin, which supports the cytokine response, and the modulation of adaptive immunity, and has antimicrobial effects that act on the respiratory system. In addition, chronic lung disease is associated with the presence of matrix metalloproteinase proteins (MMPs) which are present in higher quantities when vitamin D levels are low [13]. The absence and increased presence of these substances underlie some of the additional protective effects of vitamin D on the respiratory system. Vitamin D interacts with ACE-2 receptors [47]. The ACE-2 receptors present on the epithelial lining of the lungs, gut, and mucous membranes are well-known to be the mode of cell entry for the SARS-CoV-2 virus [18]. Low vitamin D levels are also associated with diabetes [8,19,20,26]. The pancreatic beta islet cells which contain vitamin D receptors respond to the presence of vitamin D by increasing insulin production [8,26]. Low vitamin D levels are associated with certain cancers [22]. The protective effect on certain cancers may be based on vitamin D’s role in the activation of intracellular gene transcription and protein production [22] and in the suppression of abnormal cell proliferation [23]. These mechanisms are a sample of cellular processes requiring vitamin D. Significant research has been conducted and is available in the literature that shows strong associations between vitamin D insufficiency and disease across the globe. This worldwide problem affects healthcare costs and healthcare utilization.

### 4.3. Methodological and Future Research Considerations

The strength of the article is that data were collected in the context of a pragmatic clinical trial rather than an observational study. A limitation of this study was that complete data were not available for either the intervention or the control group, as claims data were only obtainable for those subjects who were insured by the hospital healthcare plan. The two groups insured by this plan demonstrated similar demographic and co-morbidity characteristics (Table 2).

Future research using claims data can be useful in confirming or refuting that daily vitamin D3 intake at 5000 IU can reduce healthcare costs and utilization. Such studies may be particularly helpful when conducted in the context of large employment entities such as university healthcare systems, large corporations, health insurance companies, and health maintenance organizations, to help ensure the generalizability of results. Randomized placebo-controlled trials can be ethically problematic as observational studies indicate some groups are more likely to have low vitamin D levels and would be more vulnerable to diseases associated with lower vitamin D levels, as is the case of COVID-19 [8,13,14,19,20,22,25,48]. Another research possibility is the use of large cohort studies with propensity score matching. Future research also needs to consider other micronutrients that may potentiate the benefit of vitamin D3 such as magnesium. Magnesium participates in the activation of vitamin D. All enzymes involved in vitamin D metabolism appear to require magnesium [49]. National Health and Nutritional Examination Surveys data indicated a positive correlation between vitamin D sufficiency status and high magnesium intake [50].

Also, to be considered are quasi-experimental designs, which are described as non-randomized pre–post-intervention studies. Such studies are often used in medical informatics studies. An interrupted time series design is a very strong quasi-experimental design [51]. In the interrupted time series, multiple measurements are made at equal intervals in the pre- (or baseline) period which is then interrupted by the intervention period. In the post-period, multiple measurements of equal intervals are again performed. Thus, regression to the mean is controlled for and statistical analysis of means and slopes of curves can performed. The addition of a control group would further increase the strength of the design. The use of claims data would allow for daily measurements of cost and utilization during the entire study period.

In situations in which information regarding vitamin D3 intake or levels is available, pre- and post-healthcare cost and utilization levels can be compared. For example, in a group physician practice that monitors vitamin D levels in all patients and recommends supplementation to reach levels of vitamin D between 40 and 60 ng/mL, one can, via claims data, measure pre- and post-levels of healthcare costs and utilization. In addition, comparisons of healthcare costs and utilization can be made using matched controls.

## 5. Conclusions

In conclusion: 5000 IU of vitamin D3 taken daily reduced hospitalizations for any reason, emergency room visits for any reason, ICU admissions for any reason, and hospitalizations due to COVID-19 over a 10.9-month time span. Adequately powered studies of longer duration are recommended. 

## Figures and Tables

**Table 1:** Exclusion criteria.

History of hypercalcemia History of nephrolithiasis History of intolerance to vitamin D3 supplements Use of calcium at a dose > 600 mg/day (individuals using a dose greater than 600 mg of calcium per day were asked to limit the amount to 600 mg unless they had been directed by their physician to take more than 600 mg/day. If the latter was true, the potential subject was excluded from the study.) Use of vitamin D at a daily dose > 5000 IU * Use of aluminum-containing phosphate binders in patients with renal failure Use of calcipotriene Use of digoxin Use of thiazide diuretics if using: - Hydrochlorothiazide at a daily dose > 37.5 mg - Indapamide at a daily dose > 1.25 mg - Chlorthalidone at a daily dose > 12.5 mg - Metolazone at a daily dose > 2.5 mg - Methyclothiazide at a daily dose > 2.5 mg - Chlorothiazide at a daily dose > 250 mg - Metolazone at a daily dose > 0.5 mg - Bendroflumethiazide at a daily dose > 2.5 mg - Polythiazide at a daily dose > 1 mg - Hydroflumethiazide at a daily dose > 25 mg Conditions that are associated with a risk of modified vitamin D metabolism Known allergy to wool Current enrollment in another study Life expectancy < 1 month at time of screening Inability to provide informed consent Pregnant or trying to become pregnant Employee is team member in the present study

* If potential participants were found to be using vitamin D supplementation upon screening at a daily dose ≤ 5000 IU/day, they were eligible for participation by switching to the study dose. If potential participants were taking a multiple vitamin or calcium supplement and there was less than or equal to 800 IU vitamin D in it, they could continue the multivitamin or calcium supplement along with taking the study vitamin D3. Total vitamin D could not exceed 5800 IU per day combined with any supplements that contained vitamin D. Use of vitamin D at a daily dose > 5000 IU at the direction of a physician was an exclusion criterion. If a potential subject used over-the-counter vitamin D not directed by a physician at a daily dose > 5000 IU, they were eligible to participate by switching to the lower study dose.

**Table 2:** Demographic and clinical characteristics of the vitamin D supplementation and control groups.

	Vitamin D3	Control	Standardized
	(*n* = 196)	(*n* = 444)	Difference
**Age** at enrollment in years, mean ± SD	47 ± 12	50 ± 13	0.23
**Gender, *n* (%)**			
Man	46 (23)	106 (24)	0.01
Woman	149 (76)	337 (76)	0
Other	1 (0.5)	1 (0.2)	0.05
**Race, *n* (%)**			
American Indian/Alaska Native	1 (0.5)	1 (0.2)	0.05
Asian	9 (5)	26 (6)	0.06
Black/African American	23 (12)	41 (9)	0.08
Native Hawaiian/other Pacific Islander	2 (1)	0 (0)	0.14
White	144 (73)	348 (78)	0.12
More than one race	6 (3)	14 (3)	0.01
Other	11 (6)	14 (3)	0.12
**Ethnicity, *n* (%)**			
Hispanic or Latino	22 (11)	22 (5)	0.23
Not Hispanic or Latino	174 (89)	419 (95)	0.23
**Body mass index** in kg/m^2^, mean ± SD	30 ± 6	29 ± 7	0.17
**Comorbidities, *n* (%)**			
Cardiovascular disease	48 (24)	130 (29)	0.11
Respiratory disease	32 (16)	84 (19)	0.07
Eye disease	9 (5)	14 (3)	0.07
Gastrointestinal disease	79 (40)	169 (38)	0.05
Urological disease	14 (7)	52 (12)	0.16
Liver disease	3 (2)	6 (1)	0.02
Hematological disease	23 (12)	40 (9)	0.09
Dermatological disease	35 (18)	57 (13)	0.14
Diabetes	13 (7)	36 (8)	0.06
Endocrine disease (other)	28 (14)	58 (13)	0.04
Malignant disease	11 (6)	24 (5)	0.01
History of vitamin D deficiency, *n* (%)	47 (24)	137 (31)	0.15
Previous COVID-19, *n* (%)	12 (6)	19 (4)	0.08

**Table 3:** Standardized costs by treatment groups in US dollars.

	Control (*n* = 1958)		Intervention (*n* = 196)		Difference	95%-CI	*p*-Value
	Mean (SD)	Median (Q1, Q3)	Mean (SD)	Median (Q1, Q3)			
Total billed charges for any reason	69.3 (179)	20.1 (6.7, 61.9)	61.3 (103)	22.2 (8.5, 74.1)	−8.04	−24.5 to 8.4	0.36
Cost of hospitalizations due to COVID-19	0.56 (12.5)	0 (0, 0)	0 (0)	0 (0, 0)	−0.56	−2.3 to 1.2	0.48
Cost of ICU admissions due to COVID-19	0.33 (8.9)	0 (0, 0)	0 (0)	0 (0, 0)	−0.33	−0.72 to 0.06	0.58
Cost of ventilator use due to COVID-19 (zeros entry)							
Medical pharmacy prescription costs for any reason	6.4 (92.4)	0 (0, 0)	7.4 (51.4)	0 (0, 0)	1.05	−7.3 to 9.4	0.52
Freestanding prescription costs for any reason	9.05 (38.3)	1.4 (0.2, 5.4)	13.4 (34.9)	1.6 (0.4, 8.1)	4.4	−1.2 to 9.9	0.07

SD—standard deviation; CI—confidence interval. Difference uses control group as reference.

**Table 4:** Comparisons of utilization between control and intervention groups.

	Control (*n* = 1958)		Intervention (*n* = 196)		Relative Rate	95%-CI	*p*-Value	Rate Difference	95%-CI	*p*-Value
	Sum of Events or Units	Rate Per 1000 Person-Days	Sum of Events or Units	Rate Per 1000 Person-Days						
**Number of hospitalizations for any reason ^P^**	110	0.19	0	1.46 × 10^−11^	7.8 × 10^−11^	0 to N/A	0.99	−0.19	−0.21 to −0.17	**<0.0001**
**Number of ICU admissions for any reason ^NB^**	36	0.06	0	8.11 × 10^−12^	1.33 × 10^−10^	0 to N/A	0.99	−0.06	−0.08 to −0.04	**<0.0001**
**Number of emergency room visits for any reason ^NB^**	319	0.55	11	0.29	0.53	0.27 to 1.03	0.06	−0.26	−0.46 to −0.05	**0.0131**
**Number of hospitalizations due to COVID-19 ^P^**	5	8.47 × 10^−3^	0	1.97 × 10^−12^	2.3 × 10^−10^	0 to N/A	0.99	−8.47 × 10^−3^	−0.02 to −1.05 × 10^−3^	**0.0253**
Number of ICU admissions due to COVID-19 ^P^	3	5.08 × 10^−3^	0	7.25 × 10^−13^	1.4 × 10^−10^	0 to N/A	0.99	−5.08 × 10^−3^	−1.1 × 10^−2^ to 6.69 × 10^−4^	0.08
All other outpatient units * for any reason ^NB^	20,546	34.7	1388	37.3	1.08	0.87 to 1.33	0.5	2.6	−5.2 to 10.4	0.51
Number of urgent care visits for any reason ^ZINB^	969	3.47	55	2.29	0.66	0.37 to 1.17	0.16	−1.2	−2.5 to 0.17	0.08
Number of primary care physician units ** for any reason ^NB^	5111	8.69	355	9.76	1.12	0.92 to 1.37	0.26	1.06	−0.88 to 2.9	0.28
Number of nurse practitioner units ** for any reason ^NB^	893	1.52	54	1.39	0.92	0.59 to 1.43	0.7	−0.12	−0.74 to 0.5	0.69
All other professional units * for any reason ^ZINB^	26,076	47.8	1761	51.2	1.07	0.88 to 1.31	0.5	3.4	−5.92 to 13.5	0.45
Number of medical pharmacy units *** for any reason ^NB^	1674	2.88	138	3.42	1.19	0.61 to 2.29	0.61	0.54	−1.7 to 2.76	0.64
Number of freestanding prescriptions for any reason ^ZINB^	22,286	37.4	1645	43	1.15	0.96 to 1.39	0.14	5.7	−2.3 to 13.6	0.16

Models used include Poisson (P), negative binomial (NB), and zero-inflated negative binomial (ZINB). N/A refers to the upper limit of the confidence interval which could not be calculated due to the presence of zero values. The person-time denominators for the control and intervention groups were 590,348 and 37,935 person-days, respectively. Relative rate uses control group as reference; CI-confidence interval. * Unit examples: 1 unit = 1 test, such as CAT/MRI/PET scan; 1 unit = 1 treatment, such as radiation therapy; 1 unit = 1 service, such as radiology/nuclear medicine including ultrasound and imaging; 1 unit = 1 session, such as occupational/speech therapy; ** Unit examples: PCP or NP administers 3 vaccines = 3 units; ECG = 1 unit; *** 1 unit ≠ 1 medical pharmacy prescription; majority are 1 unit, but units could be based on per mg or per hour of infusion; Models used include Poisson (P), negative binomial (NB), zero-inflated negative binomial (ZINB); Outpatient includes services performed in outpatient hospital setting such as outpatient cardiologist, dermatologist, nephrologist, etc.; Primary care physician (PCP) includes general practice, internal medicine, family practice, pediatrician, and ob/gyn. Nurse practitioner (NP) includes nurse practitioner, certified registered nurse practitioner (CRNP), CRNP PCP, nurse practitioner/clinical specialist. Professional includes services performed in office setting such as cardiologist, dermatologist, nephrologist, etc. Medical pharmacy includes chemotherapy, rheumatology medications, Crohn’s medications, etc. Freestanding prescriptions include prescriptions obtained at freestanding pharmacies.

## Data Availability

The data presented in this study are available on request from the corresponding author. The data are not publicly available due to potential privacy concerns.
